# A Pilot Study of Raltegravir Plus Combination Antiretroviral Therapy in Early Human Immunodeficiency Virus Infection: Challenges and Lessons Learned

**DOI:** 10.1089/biores.2015.0038

**Published:** 2016-01-01

**Authors:** Ann C. Collier, Tae-Wook Chun, Janine Maenza, Robert W. Coombs, Kenneth Tapia, Ming Chang, Claire E. Stevens, J. Shawn Justement, Danielle Murray, Joanne D. Stekler, James I Mullins, Sarah E. Holte

**Affiliations:** ^1^ Division of Infectious Diseases, Department of Medicine, University of Washington, Seattle, Washington.; ^2^ Laboratory of Immunoregulation, National Institute of Allergy and Infectious Diseases, National Institutes of Health, Bethesda, Maryland.; ^3^ Department of Laboratory Medicine, University of Washington, Seattle, Washington.; ^4^ Department of Global Health, University of Washington, Seattle, Washington.; ^5^ Department of Neurology, University of Washington, Seattle, Washington.; ^6^ Department of Microbiology, University of Washington, Seattle, Washington.; ^7^ Program in Biostatistics and Biomathematics, Division of Fred Hutch, Department of Biostatistics, University of Washington, Seattle, Washington.

**Keywords:** antiretroviral therapy, integrase inhibitor, primary HIV, reservoir

## Abstract

Availability of integrase strand transfer inhibitors created interest in determining whether their use would decrease persistently infected cell numbers. This study hypothesized that adding raltegravir (RAL) to standard antiretroviral therapy (ART) would decrease human immunodeficiency virus (HIV)-infected CD4^+^ T cells more than standard combination ART. This was a pilot, randomized study comparing open-label standard triple ART to standard triple ART plus RAL over 96 weeks in ART-naive adults with early HIV infection. The primary objective was to compare quantity and trajectory of HIV DNA. Eighty-two persons were referred. A diverse set of reasons precluded the enrollment of all but 10. Those who enrolled and completed the study had an estimated median duration of HIV infection of 74 days at ART start. The groups had similar baseline characteristics. The RAL group had more rapid first phase plasma HIV RNA decay (0.67 log_10_ copies/mL/day) than with combination ART (0.34 log_10_copies/mL/day), *p* = 0.037. Second phase HIV RNA decay, residual viremia, cell-associated RNA, HIV DNA, CD4^+^ T-cells with replication-competent virus, and 2LTR circle levels did not differ between groups. Among those with entry plasma HIV RNA levels above the median, 2LTR circles were significantly lower over time than in those with lower entry HIV RNA levels (*p* = 0.02). Our results suggest homogeneity of responses in cell-associated RNA, HIV DNA, CD4^+^ T-cells with replication-competent virus, and 2LTR circles with early HIV in both ART groups. The kinetics of 2LTR DNA did not reflect the kinetics of plasma HIV RNA decline following ART initiation.

## Introduction

Availability of the first integrase strand transfer inhibitor created interest in determining whether its use would decrease the number of cells persistently infected with human immunodeficiency virus (HIV) type one. Raltegravir (RAL, Isentress^®^) added to standard combination antiretroviral therapy (ART) in persons with chronic HIV decreased latently infected, resting CD4^+^ T cells and had favorable effects on ileal cell-associated unspliced HIV RNA in CD4^+^ T cells^1,2^; other studies showed no virologic impact of RAL intensification.^3–6^ No difference in HIV RNA or DNA was seen in randomized studies of 5-drug RAL-containing ART versus three-drug ART in recent HIV infection.^7,8^ Persons who start ART earlier versus later have lower HIV DNA burden.^9–11^ We performed a pilot study to evaluate impact of RAL in addition to standard three-drug ART in persons with early HIV infection on virologic measures and describe the challenges and lessons learned.

## Materials and Methods

### Study participants

Participants were ≥18 years old, ART-naïve, had HIV RNA ≥500 copies/mL within 14 days before entry and had early HIV infection defined as follows: A current positive HIV EIA and western blot with either a negative HIV EIA within the past 6 months or a negative point-of-care HIV test or a nonreactive less-sensitive (LS) HIV EIA within the past month. Exclusion criteria included pregnancy, breastfeeding, and prior HIV vaccines. Complete entry criteria are listed on ClinicalTrials.gov number NCT00781287. This study was approved by University of Washington (UW)'s Human Subjects Review Committee. Participants provided written informed consent.

### Study design and procedures

This was a pilot randomized study comparing open label standard triple ART to standard triple ART plus RAL (hereafter called RAL group). We hypothesized that adding RAL to standard ART would decrease HIV-infected CD4^+^ T cell number more than standard ART. Standard ART, prescribed by the subject's primary care provider, was two nucleoside reverse transcriptase inhibitors (NRTIs) and a non-NRTI or a protease inhibitor. The study provided RAL 400 mg twice daily for up to 96 weeks. The study statistician performed the 1:1 randomization.

Participants underwent evaluations at entry, day 3, weekly for 4 weeks, at weeks 8, 12, 16, 24, and every 12 weeks until week 96. Consenting subjects underwent leukapheresis at entry, weeks 48 and 96 for assessment of infectious HIV in resting CD4^+^ T cells. Assays were performed by laboratory personnel blinded to treatment. Plasma HIV RNA levels were determined in Seattle until viral suppression using the Abbott m2000sp/rt HIV-1 RealTime assay (Abbott Molecular) and subsequently, in Bethesda using the COBAS^®^ Ampliprep/COBAS Taqman HIV-1 Test, version 2.0 (Roche Diagnostics) done in triplicate as previously described^12^ with a limit of detection of 20–48 copies/mL. Residual plasma viremia (<20 HIV RNA copies/mL) (by low copy assay) was determined by averaging Ct values obtained in triplicate from plasma specimens. Frequency of CD4^+^ T cells carrying HIV proviral DNA was determined by real-time polymerase chain reaction (PCR).^12^ For detection of cell-associated unspliced HIV-1 RNA, 500 ng of total cellular RNA was subjected to real-time PCR using TaqMan RNA-to-C_T_ 1-Step Kit (Applied Biosystems) and HIV-specific primers and probe.^13^ Frequency of CD4^+^ T cells producing infectious (replication competent) HIV was measured by quantitative coculture assays.^14^ Extra-chromosomal 2LTR circular DNA was assessed on stored peripheral blood mononuclear cells using a modified assay procedure.^4,15^ The quantity of 2LTR circles was reported as log_10_copies/10^6^ CD4^+^ T cells. CD4^+^ T cells and routine toxicity assessments were performed using standard methods by UW Department of Laboratory Medicine clinical laboratories. Toxicities were graded by standard tables.^16^

### Statistical considerations

The primary objective was to compare quantity and trajectory of HIV-infected CD4^+^ T cells, assessed by HIV DNA. Secondary endpoints included levels and trajectory of plasma HIV RNA, cell-associated RNA, quantity and half-lives of CD4^+^ T-cells with infectious virus, and clinical and laboratory toxicities. Residual plasma HIV viremia and 2LTR circles were added as secondary endpoints when these assays became available.

The study originally had a sample size of 10 per arm and 88% power to detect a 0.7 log_10_ difference in HIV DNA at 96 weeks, assuming a standard deviation (SD) of 0.5 log_10_ and using a two-sided *t*-test with alpha of 0.05. However, due to slow enrollment, the study was terminated prematurely. With five subjects per arm and an SD of about 0.2727, we would have 88% power to detect a difference of 0.54 log_10_copies/mL. We used Wilcoxon rank-sum and Fisher's exact tests to compare continuous and categorical baseline characteristics. To evaluate differences in endpoints at single time points and changes between time points, two sample *t*-tests were used. To evaluate longitudinal levels, linear mixed effects models were used to account for repeated measures. All analyses used SAS v9.3 (SAS Institute, Inc.). Estimates of first and second nonlinear viral decay rates used the model developed by Perelson et al.^17^ and nonlinear random effects to account for repeated measures.

## Results

### Enrollment, participant characteristics, and follow-up

**FIG. 1.**
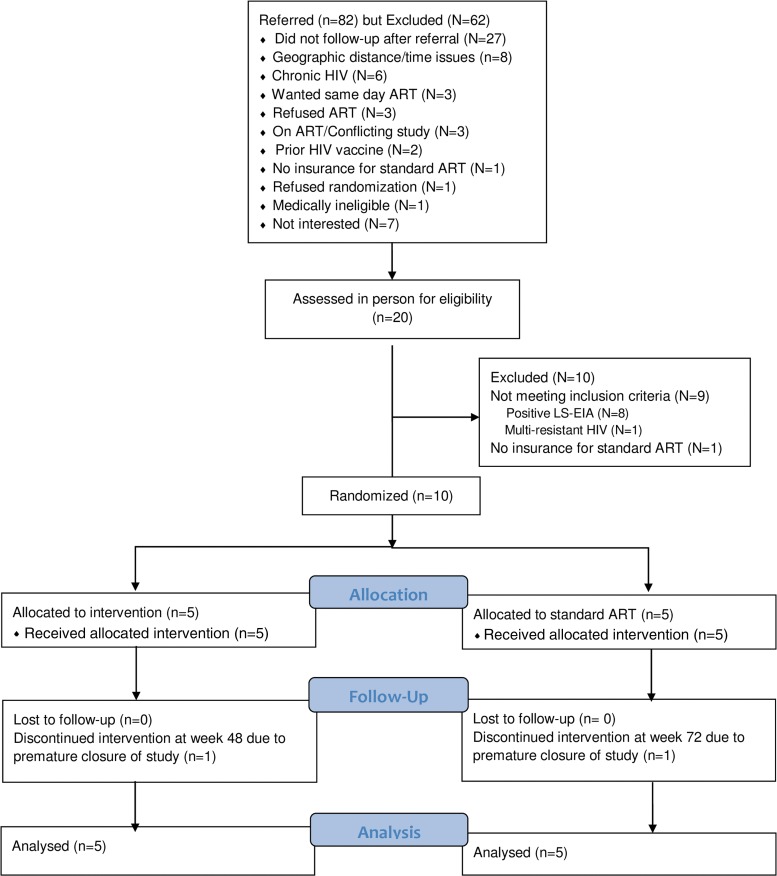
Disposition of individuals who screened for and participated in the study. ART, antiretroviral therapy; HIV, human immunodeficiency type 1; LS-EIA, low-sensitivity enzyme immunoassay.

Subjects enrolled from March 2009 to 2011. Follow-up ended in February 2012. Figure 1 describes the disposition of individuals referred for participation and enrollees.

Table 1 describes baseline characteristics of enrollees. Participants had symptoms consistent with primary HIV infection; nine sought medical attention. Each had early HIV infection with an estimated median HIV infection duration of 74 days at ART start. Each group's characteristics were similar except for a trend toward higher HIV RNA values at screening in the RAL group (*p* = 0.047). No participants were lost to follow-up. Eight subjects completed 96 weeks. Two had follow-up truncated when the study ended; one completed 72 weeks and the other 48 weeks. All but one subject were adherent to treatment; one in the RAL group stopped ART 5 days before week 96's leukapheresis.

**Table 1.** **Baseline Characteristics of Participants**

Characteristics	Combination ART (*n* = 5)	Combination ART + raltegravir (*n* = 5)	Total (*n* = 10)
Age, years, median (range)	26 (21–32)	31 (22–43)	27 (21–43)
Male, No.	5 (100)	5 (100)	10 (100)
Race, No.			
Nonhispanic white	3	5	8
Hispanic or nonwhite	2	0	2
HIV risk factor, No.			
MSM	5	5	10
Symptoms with 1° HIV, No.			
Mild (no medical attention)	1	0	1
Mild (sought medical attention)	0	2	2
Severe	4	3	7
Fiebig stage at screening, No.			
V^a^	2	4	6
VI	3	0	3
Not available	0	1	1
CCR5 delta-32 mutation status, No.			
Wild-type	5	3	8
Heterozygote	0	2	2
Baseline CD4^+^ cells/mm^3^, median (range)	432 (412–722)	690 (432–980)	511 (412–980)
Baseline CD8^+^ cells/mm^3^, median (range)	996 (572–1,499)	849 (471–1,829)	923 (471–1,829)
Baseline CD4:CD8, median (range)	0.55 (0.37–0.76)	0.92 (0.24–1.15)	0.67 (0.24–1.15)
Screening HIV RNA, log_10_copies/mL, median (range)	4.26 (2.97–5.06)	5.25 (4.42–5.89)	4.78 (2.97–5.89)
Baseline HIV RNA, log_10_copies/mL, median (range)	4.26 (2.20–4.92)	5.21 (3.90–6.00)	4.28 (2.20–6.00)
Baseline HIV RNA (low copy assay)log_10_copies/mL, median (range)	4.43 (3.20–5.09)	4.86 (4.05–5.89)	4.48 (3.20–5.89)
Baseline HIV cell-associated RNA, log_10_copies/mL, median (range)^b^	3.19 (2.65–3.40)	3.21 (2.83–4.48)^3^	3.19 (2.65–4.48)
Baseline HIV DNA, log_10_copies/10^6^ CD4^+^ T-cells, median (range)	3.37 (3.01–3.65)	3.57 (3.12–4.05)	3.47 (3.01–4.05)
Baseline infectious virus, log_10_infectious units/10^6^ PBMCs, median (range)^c^	61 (8–421)	157 (41–323)	82 (8–421)
2-LTR circles log_10_copies/10^6^ CD4^+^ T-cells, median (range)	77 (3–224)	25 (0–146)	25 (0–224)
Days from estimated date of infection to ART start, median (range)	69 (44–98)	78 (56–83)	74 (44–98)
First ART regimen, No.			
NNRTI-based	2	2	4
PI-based	3	3	6

^a^ Two subjects had Fiebig I/II at diagnosis.

^b^ One subject is missing in the raltegravir-containing group.

^c^ One subject is missing data in each group.

ART, antiretroviral therapy; CCR5, C-C chemokine receptor 5; HIV, human immunodeficiency virus; LTR, long terminal repeat; mL, milliliter; mm, millimeter; NNRTI, non-nucleoside reverse transcriptase inhibitor; No., number; PBMCs, peripheral blood mononuclear cells; 1°, primary HIV; PI, protease inhibitor; RNA, ribonucleic acid.

### Virologic, immunologic, and clinical outcomes

**FIG. 2.**
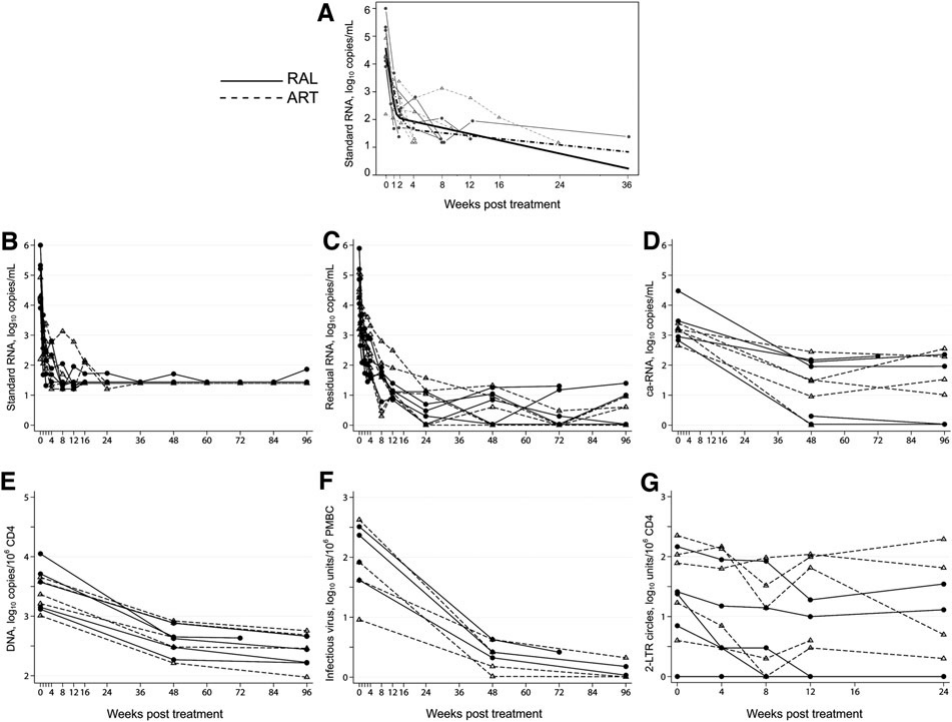
Virologic parameters by treatment group over time. **(A)** Plasma HIV RNA (log_10_ copies/mL) with two phase decay to undetectable model. **(B)** Plasma HIV RNA, log_10_ copies/mL; values below the limit of detection were set to half the limit of detection. **(C)** Residual viremia, log_10_ RNA copies/mL. **(D)** HIV Cell-associated RNA, log_10_copies/mL. **(E)** HIV DNA, log_10_ copies/10^6^ CD4^+^ T cells. **(F)** CD4^+^ T cells with replication-competent HIV, log_10_ infectious units/10^6^ peripheral blood mononuclear cells. **(G)** 2LTR circles, log_10_copies/10^6^ CD4^+^ T cells.

The RAL group had more rapid first phase plasma HIV RNA decay (0.67 log_10_copies/mL/day) than the other group (0.34 log_10_copies/mL/day), *p* = 0.037 (Fig. 2, part A). Rates of second phase decay were not significantly different in the groups. Eight subjects achieved plasma HIV RNA <50 copies/mL by week 12 of ART (part B). No differences were seen between groups in cell-associated RNA, HIV DNA (primary endpoint with a difference in means of 0.083 log_10_copies/mL), or CD4^+^ T-cells with infectious virus at 48 or 96 weeks or in change from baseline to either 48 or 96 weeks (parts D–F). Similarly, levels of residual viremia (based upon the low copy assay) (part C) from samples collected 20 or more weeks post-infection or of 2LTR circles (part G) did not differ between groups.

Given a trend toward differences between groups in screening plasma HIV RNA levels, we reanalyzed virological parameters by dividing subjects by median entry plasma HIV RNA level of 4.48 log_10_copies/mL. Among those with higher entry plasma RNA, 2LTR circle levels were significantly lower over time than in those with lower entry RNA (*p* = 0.03) (Table 2). There were no differences between high and low baseline RNA groups in cell-associated HIV RNA, HIV DNA, or numbers of CD4^+^ T-cells with infectious virus.

**Table 2.** **Comparison of Longitudinal Virologic Levels by High Versus Low Baseline HIV RNA**

Virologic parameter	Low HIV RNA, *N* = 7	High HIV RNA, *N* = 3	*p*
HIV DNA, log_10_copies/10^6^ CD4^+^ T-cells	2.92	2.77	0.46
Infectious virus, log_10_ infectious units/10^6^ PBMCs	1.15	0.77	0.39
HIV cell-associated HIV RNA, log_10_copies/mL	1.84	1.57	0.62
2LTR circles, log_10_copies/10^6^ CD4^+^ T-cells	2.05	0.85	0.03

DNA, deoxyribonucleic acid; HIV, human immunodeficiency virus; PBMCs, peripheral blood mononuclear cells; RNA, ribonucleic acid.

There were no differences between randomized groups in CD4^+^ or CD8^+^ T cell counts or CD4:CD8 ratios. No subjects developed AIDS-defining or HIV-associated illnesses. One subject had transient, self-limited grade 3 neutropenia unrelated to ART. All other signs, symptoms, and laboratory values were grade 2 or less and typical of the ART used.

## Discussion

When ART was started during early HIV, our results suggest homogeneity of responses in residual viremia, cell-associated RNA, HIV DNA, and CD4^+^ T cells with infectious virus. First phase decay of HIV RNA was faster in subjects receiving RAL in addition to standard triple ART.

It was of interest that levels of 2LTR circles were lower over time among subjects with higher compared with lower pre-ART HIV RNA levels. We hypothesize that the difference in 2LTR kinetics represents differences in infected CD4^+^ cell clearance and different rates of replenishment between these two groups of initially viremic subjects. Some studies have suggested that 2LTR circles may increase in persons with chronic HIV when they are treated with RAL-containing regimens but others, including the present study in persons with early HIV, have not seen such changes.^11,18,19^ Following ART initiation, kinetics of 2LTR DNA did not reflect the kinetics of plasma HIV RNA decline.

Enrolling our study was unexpectedly challenging. Our group has enrolled 394 subjects with acute or early HIV since 2002 in an observational study that does not require or exclude ART; the proportion of immediate ART start has varied over time, likely reflecting evolving views of ART's potential benefits and risks. When the current study was conducted, U.S. treatment guidelines were not supportive of early ART.^20^ Other issues included the lack of sensitive, readily available assays during the study to detect early HIV; eight potential participants with recent sexual HIV exposure and documented past negative HIV tests (as recently as 7 months previously), suggesting recent HIV infection, had positive LS-EIA assays so were excluded from participation. A wide variety of other reasons prevented potentially eligible persons from enrolling.

Limitations of this pilot study include being underpowered for treatment comparisons, selection bias toward participants with symptomatic primary infection, and lack of subject diversity (although reflective of the epidemic in the Pacific Northwest). Nonetheless, with increased interest in early ART and understanding relationships among different virologic parameters, the lessons learned from this study may increase knowledge about effects of ART in early HIV and help the design and implementation of future studies.

## Conclusions

A diverse set of reasons adversely impacted enrollment in this pilot study. The RAL group had more rapid first phase decay than combination ART without RAL. Our results suggest homogeneity of responses in cell-associated RNA, HIV DNA, CD4^+^ T-cells with replication competent virus, and 2LTR circles to early ART in both treatment groups. Following ART initiation, the kinetics of 2LTR DNA did not reflect the kinetics of plasma HIV RNA decline.

## Abbreviations Used

1°: primary HIV
ART: antiretroviral therapy
CCR5: C-C chemokine receptor 5
HIV: human immunodeficiency virus
LS: less-sensitive
LTR: long terminal repeat
mL: milliliter
mm: millimeter
NNRTI: non-nucleoside reverse transcriptase inhibitor
No.: number
NRTIs: nucleoside reverse transcriptase inhibitors
PBMCs: peripheral blood mononuclear cells
PCR: polymerase chain reaction
PI: protease inhibitor
RAL: raltegravir
RNA: ribonucleic acid
SD: standard deviation
